# Using Intervention Mapping to Develop, Implement, and Evaluate a Community Engaged Crime Reduction Intervention

**DOI:** 10.1002/jcop.70109

**Published:** 2026-07-01

**Authors:** Catanya G. Stager, Samantha Whitfield, Ariann Nassel, Henry Irby, Mike Wood, Tiffany Osborne, Grace Okoro, Melissa Thompson, Penny Willis, Dwayne A. Crawford, Matthew Kiefer, Erin Carley, Christson Adedoyin, Yu‐Mei Schoenberger, Lori B. Bateman

**Affiliations:** ^1^ Heersink School of Medicine University of Alabama at Birmingham Birmingham Alabama USA; ^2^ School of Public Health University of Alabama at Birmingham Birmingham Alabama USA; ^3^ Jefferson County Sheriff's Office Birmingham Alabama USA; ^4^ Helena Police Department Helena Alabama USA; ^5^ National Organization of Black Law Enforcement Executives Birmingham Alabama USA; ^6^ Lions Clubs International Foundation Oak Brook Illinois USA; ^7^ Department of Social Work Samford University Birmingham Alabama USA

**Keywords:** adolescents, community‐based participatory research, crime, focus groups, police, social cohesion, violence

## Abstract

This study aimed to develop, implement, and evaluate a multi‐level violence prevention initiative codesigned with the community in Bessemer, Alabama. Guided by a community‐based participatory research approach, we developed “Building a Better Bessemer,” a holistic intervention targeting violent crime at the socio‐ecological levels (individual, relational, community, and societal levels) through intervention mapping. The intervention included youth social‐emotional learning, mentoring, parental support, police engagement, and community improvement projects. Evaluation included pre‐post surveys and focus groups. Among youth self‐reported outcomes, significant improvements were seen in social cohesion (*p* = 0.009) and decreases in conduct problems (*p* < 0.001) and total difficulties (*p* < 0.003). Focus group results reported strong support for the program's content and delivery. Community‐driven, multi‐level violence prevention interventions are feasible and effective in communities with high crime and show promising outcomes in youth behavior and community safety.

## 1 Introduction

Gun‐related fatalities are now the leading cause of death overall for adolescents and young adults in the U.S. (CDC 2012), with the most current data indicating that firearm‐related deaths have surpassed motor vehicle deaths for all ages (Goldstick et al. 2022). Furthermore, community violence is concentrated in economically disadvantaged and minoritized communities (CDC 2024; Fowler et al. 2017; Zimmerman and Messner 2013), with stark community disparities observable. Compared to white males aged 15‐34, for Non‐Hispanic Black males face a firearm homicide rate 24 times greater, and an overall homicide rate 18 times higher (CDC 2009, 2012; Villarreal et al. 2024). Therefore, addressing community violence in these communities, with a focus on reducing crime rates and increasing healthier outcomes, remains a public health imperative.

### 1.1 Community Violence and Social Determinants of Health

Community violence is influenced by social determinants of health (SDH) (Hahn 2021), such as increased neighborhood disorder, socioeconomic deprivation, inadequate police resources, and low social cohesion (Armstead et al. 2021; Ross and Jang 2000; Yonas et al. 2007). Low social cohesion, for example, is linked to risk factors for youth violence, including substance abuse, delinquent behavior, and negative perceptions, such as fear of victimization and lack of safety (Bateman et al. 2017), as well as self‐reported health quality (Baum et al. 2009). Alternatively, positive perceptions of social cohesion (Forrest and Kearns 2001) and positive family factors can help decrease the likelihood of violence (Logan et al. 2016) and protect against community violence outcomes for youths exposed to violence (Hardaway et al. 2016).

Community violence is not only an outcome of SDH, but it also serves as an SDH itself. In this way, community violence can influence disease risk and later downstream disease outcomes through upstream perceptions and behaviors (Diez Roux and Mair 2010). Exposure to community violence, as victims or witnesses, and particularly for children, can lead to systemic physiological, neurological, and psychological dysfunction across sex, gender, and age (CDC 2024; Coulon et al. 2016; Fagan and Catalano 2013). Community violence is associated with poorer health behaviors, including cigarette smoking, illicit drug addiction, and poor sleep, and negative health outcomes such as increased incidence of sexually transmitted infections, obesity, asthma, Type II diabetes, and cardiovascular disease (Akinboboye et al. 2022; Semenza et al. 2024; Theall et al. 2022). The stress‐inducing environment of a community with a high rate of violent crime can also contribute to poor mental health outcomes related to anxiety and depression, whether individuals experience violence directly or only perceive it (CDC 2024; Coulon et al. 2016; Fagan and Catalano 2013; Fowler et al. 2009).

Despite the wealth of research on risk factors and prevention, little research has examined community‐engaged solutions to address youth violence from the viewpoint of community members and stakeholders who represent violence‐prevalent communities (Ross et al. 2021). However, studies utilizing community‐engaged research, such as community‐based participatory research (CBPR), show promising findings (Kingston et al. 2021; Ross et al. 2021). CBPR approaches can effectively reduce the impact of community violence with the implementation of preventive approaches to community violence (Stalker et al. 2020).

This paper describes the community‐engaged design, implementation, and feasibility outcomes of a community violence reduction intervention, *Building a Better Bessemer: Innovations in Community‐Based Crime Reduction (BBB)*. The final BBB intervention components and outcomes were defined by community‐driven priorities, and BBB was developed through a comprehensive community‐engaged process supported by a Department of Justice Bureau of Justice Assistance (DOJ BJA) funded grant in which institutional partners, the National Organization of Black Law Enforcement Executives (NOBLE) and the University of Alabama at Birmingham worked with the community to co‐design and implement the intervention.

## 2 Materials and Methods

This study was approved by the Institutional Review Board of the University of Alabama at Birmingham, number 300005650. Written informed consent was obtained from parents/guardians, and written assent was obtained from youth/children.

### 2.1 Study Population

Bessemer, located in Jefferson County, Alabama, has a population of 26,019, 66.6% Black, 21.6% White, 10.4% Hispanic/Latino, and 4.3% mixed race according to the most recent US Census data (U.S. 2022). Bessemer led the nation in violent crime for U.S. cities with a population of 25,000 or more in 2021–2022, and in 2022, the violent crime rate in Bessemer was 13.87 per 1000 residents, 145% higher than Alabama's rate (5.66/1000) and 37% higher than Jefferson County's rate (10.09/1000) (Hudnall et al. 2024). In 2023, Bessemer was labeled the most dangerous city in America (NeighborhoodScout's Most Dangerous Cities 2023 2023), with a 28.1% poverty rate, 22% of residents earning <$20,000 per year, 17% of residents without a high school diploma by age 25, and only 31% of graduates considered college and career ready (Spencer 2022). (See Table 1.) Despite these challenges, Bessemer City developed and is implementing a strategic plan with a goal toward developing a “livable” city where all community members “can find opportunities” (Services 2020).

**Table 1:** Social and demographic values, comparison of Jefferson County and the city of Bessemer.

	Jefferson county	Bessemer
Race/Ethnicity		
Population (*n*)	674,721	26,019
Black (%)	43.1%	66.6%
White (%)	52.8%	21.6%
Hispanic/Latino (%)	5.9%	10.4%
Mixed Race (%)	1.6%	4.3%
Social Demographics		
Overall Crime Rate (per 1000)	40.50	87.0
Violent Crime Rate (per 1000)	4.81	13.87
Poverty Rate (% in poverty)	14.2%	28.1%
Residents Earning < $25,000 (%)	23.1%	38.8%
Mean household income ($)	$66,388	$39,613
Residents with no diploma by age 25 (%)	11%	17%
Career College Readiness (%)	63%	31%
Graduation rates (%)	92%	67%

### 2.2 Community‐Engaged Research Approach

Our community‐engaged research approach was guided by CBPR principles, which served as the framework for developing the project and directing the priorities of its programs. CBPR empowers community members to address their own concerns while equitably involving diverse partners in all phases of research and allows community partners and academic partners to work together equally to address locally defined priorities and solutions (Israel et al. 2017).

BBB has been built with community trust, partnerships, and infrastructure already established through NOBLE's local chapter and the UAB Minority Health & Health Equity Research Center (MHERC) Bessemer Building Healthy Communities (BHC) Coalition. The MHERC's partnership with the Bessemer community dates back to 1998, and the Bessemer BHC Coalition was established in June 2018 with 32 members representing residents and community stakeholders to prioritize and address important issues. The coalition was eager to be engaged in this project and was foundational to creating a cross‐sector partnership in support of the BBB initiative.

### 2.3 Intervention Mapping

As part of the community‐engaged research approach, we used intervention mapping (IM) to design, implement, and evaluate a violence prevention intervention with community collaboration at each step of the process. IM is a systematic means of integrating theory, research, and collected data from a target population to promote structured planning to develop, implement, and evaluate an intervention (Bartholomew et al. 1998; Eldredge et al. 2016). IM has been used in health promotion program planning to provide an ecological approach to plan, implement, and assess an intervention (Eldredge et al. 2016). Central to the development of IM is first the identification of the health issue, which is then followed by the development of the intervention, based on current social and behavioral theories. We implemented the project through a six‐step process: (1) conduct needs/assets assessment, (2) develop program objectives, (3) select theory‐based methods and applications, (4) design the intervention, (5) adopt and develop implementation plan, (6) create intervention evaluation (Bartholomew Eldredge et al. 2016; Fernandez et al. 2019). For this project, during a 12‐month planning phase that included individual interviews, focus groups, and surveys with members of the Bessemer community, as well as geographic information systems (GIS) mapping, we designed and refined the final intervention using IM.

#### 2.3.1 Step 1: Conduct Needs/Assets Assessment

Based on longstanding partnerships through the UAB MHERC, in February 2021, we established a local cross‐sector Partnership Advisory Board (PAB), which became an integral part of the BBB Team, and the PAB served as a board of advisors tasked with oversight and accountability of the BBB efforts. Aimed at building capacity and promoting sustainable collaboration with local community‐based organizations (CBOs) and community stakeholders, the PAB met monthly with institutional partners to guide the design, implementation and evaluation of the project and to ensure sustainability, as well as that the community, with its unique history and culture, was appropriately represented.

Our community‐engaged needs assessment included a literature review of evidence‐based crime prevention strategies, interviews with community stakeholders, and GIS mapping to identify local crime hot spots. Through one‐on‐one interviews with stakeholders (law enforcement, community leaders, victims of crime, civic leaders, and residents, *N* = 18), we identified community perceived causes, outcomes, and solutions to reduce community violence (Stager et al. 2024), as detailed in a previous manuscript, Overall, interviews indicated violent crime was perceived as being primarily committed by people 30 and under, and perceived causes of violent crime included widespread poverty, gang affiliation, and poor parenting. Concerns about youth in illegal activities and mistrust between law enforcement and residents contributed to decreased social cohesion and increased law enforcement presence, all of which result in a poor reputation for the city. To address crime and reduced trust between youth and law enforcement, robust community engagement and community resources were seen as needed. Participants suggested that solutions should include law enforcement as partners and that programs should focus on young people, parenting, and conflict resolution (2024).

To identify specific areas in Bessemer to focus the intervention plan, referred to as the Action Plan, we used a place‐based mapping approach (Hites et al. 2013) of violent crime in Bessemer from 2017 to 2021 (Quarter 1) to determine the highest density of crime for the time period available. Violent crime incidence data (incidents of assault, domestic violence, kidnapping, robbery, rape, theft of a vehicle, burglary, and discharge of firearms) from January 2017 to May 2021 were obtained from the City of Bessemer Police Department's (BPD) Computer Aided Dispatch (CAD) system. The mapping, which provided a contextual and geographic sense of the highest crime incidents, utilized Geographic Information System (GIS) (Esri ArcGISPro 2.8) and crime incidence data. Geospatial mapping of crime data identified “hotspots” of the violent crime data to better understand the crime in relation to the city and the study areas. The mapping indicated that the highest incidents of violent crime occurred in and around Bessemer public housing communities, which was where the BBB intervention was then focused.

#### 2.3.2 Step 2: Develop Program Objectives

The stakeholder interview findings and GIS results were used, in conjunction with evidence‐based approaches documented in peer‐reviewed literature, to co‐design an initial intervention plan guided by feedback from community interviews. The research team shared the interview analysis iteratively with the PAB at each stage of the process, and in monthly meetings, worked with the PAB to co‐design an initial Action Plan. The PAB helped identify individuals to provide feedback on the initial Action Plan, and the PAB recommended community organizations as program partners.

Community feedback indicated widespread fear over violent crime was prevalent, and minoritized youth under age 30, who have poor relationships with law enforcement, were perceived to be driving criminal activity. Based on the community‐driven priorities, the BBB team designed a multi‐level intervention plan with objectives to: (1) implement programming that focused on preventing high‐risk youth from becoming involved in crime; (2) focus programming in housing communities identified as hot spots in the GIS analysis; (3) target adults through built and social environment programs, such as citizen town halls, the development of neighborhood association plans, and beautification of public spaces. Developing the program objectives and intervention activities based on the local feedback reflects emphasis on the local relevance of public health problems. (Israel et al. 2017).

#### 2.3.3 Step 3: Select Theory‐Based Methods

Step 3 of selecting theory‐based methods was done in conjunction with Steps 1 and 2. Based on community violence studies (Salzinger et al. 2002; Tolan et al. 2003; Yonas et al. 2010; Yule et al. 2019) and the needs assessment, the project used the *CDC Social‐Ecological Model: A Framework for Prevention (*CDC 2024 *)* (SEM) as the framework to develop the intervention. This model, derived from social ecological theories, supports an approach that includes evidence‐based interventions at multiple levels—individual, social relationships, community settings, and societal—to develop sustained preventive outcomes to address youth, parent, and law enforcement needs (CDC 2024). The model was presented to the PAB as we designed the final EAP (Step 4), and this model framed the intervention components, with solutions based on previous community‐engaged research (Stager et al. 2024).

#### 2.3.4 Step 4: Design the Intervention

**Figure 1:**
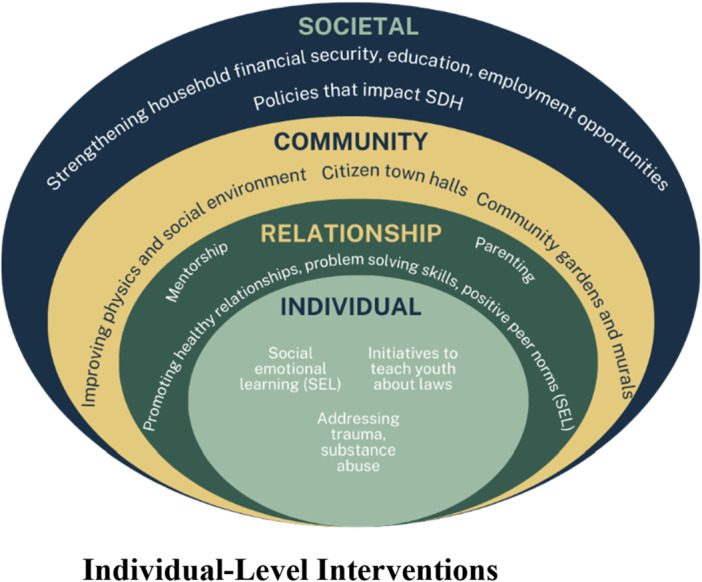
Building a better bessemer intervention: social ecological model.

Based on findings from Steps 1–3, the BBB team designed a community violence prevention intervention that targeted multiple levels of influence, in line with the CDC model (See Figure 1) and community‐engaged research principles (Israel et al. 2017). Individual components included social‐emotional learning (SEL) education (Nasheeda et al. 2019), as SEL education has been found to target skills such as critical thinking, decision making, and the development of social skills (Gargan 2017; Holahan and Batey 2019; Matischek‐Jauk et al. 2018) and reduce tobacco, illicit substance use (Nasheeda et al. 2019), and delinquency‐related behavior (Matischek‐Jauk et al. 2018). Other key components included student mentoring (Goldner and Ben‐Eliyahu 2021) as well as the NOBLE's Law and Your Community (TLYC) curriculum to teach youth about interacting with law enforcement (National Organization of Black Law Enforcement Executives 2024). Relationship level components included parent mentorship (Goldner and Ben‐Eliyahu 2021), with SEL programming and student activities to promote healthy relationships, problem‐solving skills, and positive peer norms. At the community and society levels, programs addressed improving the physical and social environment (i.e., blight reduction through community gardening and murals) (Funderburk and Powers 2018; Hume et al. 2022; Kondo et al. 2018), and programs to address community issues such as citizen town halls and the development of neighborhood associations (Fader et al. 2020). These programs were initially implemented in a 2021 summer camp called an Early Action Project (EAP) and refined for Action Plan implementation in the 2022–2023 summer camps and after‐school activities.

### 2.4 Early Action Project (EAP)

In conjunction with designing the Action Plan, we developed and implemented an Early Action Project (EAP) in the summer of 2021 as required by the funder. The EAP served to engage the community while pilot testing proposed intervention components. Our EAP consisted of a summer camp (2021) implemented by community partners, including Bessemer City Schools, as well as the Bessemer Housing Community and the Bessemer Police Department. To identify implementation partners, the research team interviewed community partners, recommended by the Bessemer BHC coalition and the PAB, about their role, operational costs, and potential effectiveness. The research team reported back the interview outcomes to the PAB for discussion and EAP refinement, based on the PAB's feedback, experience, and recommendations. The research team shared the PAB recommendations with the local Bessemer BHC Coalition. After receiving feedback from both community boards, the research team finalized the EAP, which was again presented to the PAB, to move forward in selecting candidate community partners for implementation. With the PAB approval, the research team then communicated with the selected partners their EAP roles, and community partners were given the opportunity to provide feedback on their capacity to contribute and commitment as a partner. This portion of the EAP development was protracted due to iterative meetings, contract reviews, amendments, and budgeting details. Eventually, the BBB team secured the community partners for the implementation of the EAP.

To recruit youth to participate in the EAP, community partners attended events including carnivals, Little League football games, and activities at the local recreation center to disseminate program fliers, and teachers, community center directors, and community leaders shared fliers with youth. Children (elementary school) and youth (middle and high school) participated in the EAP. During the EAP, gathering process data on participants and programming (attendance, number of partners, activities) was the focus. EAP activities included individual, relationship, community, and societal components, as described above. EAP highlights included a student mural, TLYC programming, SEL activities, and community‐partner‐led programming.

### 2.5 Individual‐Level Interventions

These programs focused on school‐age children (elementary students) and youth (middle and high school students), as the prevention of delinquency was of utmost importance to the community. Targeting the younger population was supported by focus groups and PAB feedback provided in Steps 1 and 2 and was integrated into the Action Plan as a community‐led solution to community violence in Bessemer. Community feedback indicated a consensus that youth were perceived as the primary offenders of crime, and the community solution was to develop and offer youth activities outside of school to reduce delinquent behavior and to cultivate their interests as a diversion to crime (Stager et al. 2024). The research team essentially took those community suggestions and developed interventions that provided the community with what they asked for, an example of co‐collaboration from beginning to end.

We developed a no cost summer camp and afterschool programs for children and youth to educationally address life skills (including gardening, art therapy, mentoring, field trips, physical activity and nutrition, financial literacy and academic tutoring) and social emotional learning (SEL), which focuses on communication and social skills development to reduce problematic behaviors (Gargan 2017; Holahan and Batey 2019; Matischek‐Jauk et al. 2018). The afterschool programs were 10‐week sessions, hosted at a community location, and the summer camp session was a 4‐week all‐day session (See Table 2). All sessions were no cost. Within the summer camp and afterschool programs, we utilized Lions Quest, an evidence‐based SEL curriculum (Eisen et al. 2003; Jones et al. 2017; Jones et al. 2022).

**Table 2:** Social‐emotional learning (SEL) topics for the intervention by group.

	Groups			
Week	Grade 3–5	Grade 6–8	Grade 9–12	Parents
1	Orientation –	Orientation –	Orientation –	Orientation: What is SEL and Lions Quest
2	Clarifying the Conflict (Gr4)	Expressing Emotions and Needs Constructively (Gr8)	Your Personal Stress Plan (L3)	Learning SEL Together
3	Give the Gift of Listening	The Benefits of Effective Listening	Communicating with WWWH Messages	Building Family Communication
4	My Normal Ups and Downs	Exploring Emotions	Exploring Emotions in Action	Exploring Emotions to Build Healthy Family Relationships
5	Calming and Reflecting on Emotions	My Anger Buttons	We Can Only Control our Response	Regulating Emotions to Build Healthy Family Relationships
6	My Treasure Chest of Skills and Qualities	The Three‐Legged Stool of Self‐Confidence	Reducing the Risk Factors and Building Resilience	Building Up Children with Self‐Esteem and Self‐Confidence
7	The Problem with Drugs (Gr5)	Leading the School in Healthy Choices	Building Self‐Esteem Through the Development of Positive Self‐Identity and Positive Racial Identity.	Building Self‐Esteem through the development of Positive Self‐Identity and Positive Racial Identity
8	What are the Influences (Gr 5)	Target Your Decisions (Gr8)	The Teenage Brain and Decision Making	Keeping Your Child Safe from Bullying
9	I am Right on Target	Do You Have Plans	Setting Goals and Carrying them Out	Game Changer: Organizational and Goal Setting Skill
10	Positive Thinking Achieves Goals	If You Put Your Mind to It, You Can Do	Growth Mindset	Growth Mindset

The BBB team also embedded The Law and Your Community™ education (NOBLE's nationally recognized flagship program) to address youth and law enforcement relationships (National Organization of Black Law Enforcement Executives 2024) since the interviews (see Step 1) of community members found that the TLYC program was feasible and acceptable. The BPD played a central role in the intervention as they taught the TLYC sessions and led mock police‐encounter scenarios to experientially reinforce concerns/issues important for young people to understand and comprehend. During this portion of the session, students participated in various roles along with the police. Additionally, police officers attended the programs, taught sessions on careers in law enforcement at the EAP and summer camps (for elementary and middle school students), helped create a mural, contributed to building community gardens, and volunteered in both uniform and plain clothes during the end of summer camp celebrations.

### 2.6 Relationship‐Level Interventions

Mentoring was articulated as an effective solution in Step 1, and it was included in our Intervention in our summer camp and afterschool program. Mentoring, with adult support and structured activities led by local mentoring community organizations, provided the opportunity for youth and teens to build relationships with adults and peers (Smith et al. 2018), to help children and youth reduce the risk of delinquent behaviors and build emotion‐regulating and problem‐solving skills (Frazier et al. 2019). The intervention also included an SEL program for parents, also designed by Lions Quest, since parenting challenges contribute to youth crime (Ross et al. 2021), and Lions Quest provides extensive support for family and community engagement (Jones et al. 2022).

### 2.7 Community Level Interventions

Community‐level interventions focused on both components of (1) police outreach with community engagement and (2) community spaces. The police outreach (as service learning, professional development, and positive engagement) used NOBLE's TLYC programming (Smith et al. 2021), with the goals of increasing trust, cohesiveness and informed policing. The community spaces component addressed local shared spaces with the help of community organizations, and it focused on addressing and improving green space, vacant lots, safety concerns, and access to healthy foods through community murals (Adams 2020) and community garden (Funderburk and Powers 2018; Hume et al. 2022).

### 2.8 Societal Level Interventions

To address solutions to crime at the societal level, the BBB team held neighborhood meetings and citizen town halls (Scheller and Yerena 2018), where residents were given the means to articulate concerns to the mayor/city leadership. Our intervention facilitated these forums and assisted in driving policy reform and increased partnership between police and citizens. Prior to the BBB intervention, Bessemer did not have neighborhood associations, which community members felt could be important to decreasing crime. Therefore, these meetings served as the beginning of Neighborhood Association planning and created a venue for community members to easily share information with the city and with law enforcement. These societal‐level components of the intervention reflect the long‐term process and commitment to sustainability held by community and academic partners (Israel et al. 2017).

#### 2.8.1 Step 5: Adopt and Develop the Implementation Plan

The BBB team collected process data and lessons learned in the EAP to finalize the Action Plan and develop the implementation plan. Further, in Step 5, we conducted focus groups (Fall 2021) to ensure that community voices were integrated into all aspects of the plan. Conducting focus groups at this stage in the implementation plan illustrates the cyclical and iterative nature of a community‐engaged project (Israel et al. 2017), and enabled us to gain further insight on the anticipated impact, feasibility, and acceptability of the intervention plan. Focus groups were segmented by law enforcement officers, PAB members, young black males, school system administrators, and mixed community members (2 groups). Themes that emerged (by focus group type) included: the younger generation as perpetrators (law enforcement), motivation behind young men involved in crime (young black men), schools overburdened to address crime (school system administrators), and youth committing crime (mixed group). Overall, the focus groups indicated, through strong consensus, that the intervention should focus on school‐age children to redirect their energies prior to adulthood. These findings corroborated with our interview study findings (Step 1) (Stager et al. 2024). Participants supported the proposed multi‐level intervention and suggested implementation partners. Focus groups also emphasized that trained professionals who come from the community should lead the intervention programs. Other concerns included the importance of addressing barriers to participation and the need for sustainability. The Action Plan was revised based on focus group findings.

To further develop the implementation plan, we received community feedback through a survey distributed at a local public event. Nearly 90% (*n* = 56) of survey participants (*N* = 64) agreed or strongly agreed that crime was an important issue in the community, and just under half (44%) indicated they did not feel safe. When asked to rate (scale of 1–6) the most effective programs that could reduce crime in Bessemer, 77% of community members ranked children's programs in the top 3 response categories; parenting programs were ranked second with 70%; followed by Bessemer PD leading community events (55.0%); mental health responders embedded with the BPD (54%); at‐risk youth programs like community mural painting and gardening (42%); and finally, town halls (30%).

Community members ranked trusted organizations to implement the intervention as well as intervention components, which led to the final version of the Action Plan, as well as the identification of implementation partners. Our final step included taking the Action Plan to the Mayor's office and City Council, where we were provided support for implementation. Once the Action Plan was finalized, the research team worked with the PAB and involved partners to develop the implementation plan, including timelines, financial contracts, community partner training, and detailed protocols for recruitment and programming.

#### 2.8.2 Step 6: Create Evaluation Plan

The BBB team developed a mixed‐methods evaluation plan to determine the feasibility and effectiveness of our Action Plan, including both process and outcome evaluation components. The evaluation plan was developed in conjunction with the finalization of the Action Plan, and potential outcome measures were selected by the research team, based on the intervention plan and feedback elicited from the PAB.

**Process**. We included process evaluation measures to ensure protocol fidelity and to document lessons that could be incorporated in future implementation and dissemination efforts. Understanding an intervention's success, processes, and key intermediate outcomes is important to determining the feasibility of an intervention and its effectiveness, and it is a critical step in developing intervention strategies that can be implemented widely in similar settings. We also assessed the extent to which the intervention strategies were implemented into initiatives through checklists and partner surveys. This information was collected by the UAB study staff. Process data included participant data such as attendance and demographics. Data was also gathered on the number of events held and the number of partners involved.

**Outcomes.** Program participants completed surveys to measure knowledge (for the Law and Your Community programming), self‐reported behavior (prosocial, and difficult behaviors (using the Strengths and Difficulties questionnaire (Goodman et al. 1998)), social cohesion (Dragolov et al. 2016), and collective efficacy (Sampson et al. 1997). We evaluated the outcomes of the intervention through qualitative and quantitative data analysis. Perceptions and experiences of the intervention were explored through focus groups of parents/guardians, students, and program coordinators.

**Program Surveys.** To evaluate the program feasibility and effectiveness, students and parents completed pre‐ and post‐test surveys (age‐appropriate assessments). Students included children (those in the elementary grades 3–5) and youth (those in grades 6–12, or adolescents). Adults were guardians or parents of students. Due to differences in literacy ability between elementary and middle/high school ages, students received different, age‐appropriate instruments in their respective surveys (pre‐ and post‐): children (grades 3–5) were given instruments for surveys appropriate for their younger literacy skills while youth (grades 6–12) were given survey instruments with language appropriate to their literacy ability, in accordance with the survey instrument instructions. In the analysis that follows, these results are separated by these three groups: children, youth, and adults. Elementary students (grades 3–5) completed a survey with questions from UNICEF social cohesion (Dragolov et al. 2016) (a measure for positive social relations) and the Panorama survey (Moulton and Gehlbach 2021) for measures for SEL outcomes. For middle and high school students (youth), three instruments were used: (1) UNICEF social cohesion (Dragolov et al. 2016), (2) sense of safety (NEWS‐Y) (Mujahid et al. 2007; Rosenberg et al. 2009), and (3) self‐reported behavior, including emotional problems, conduct problems, hyperactivity/inattention, peer relationship problems, and prosocial behavior (Strengths and Difficulties Questionnaire, or SDQ) (Goodman et al. 1998; Muris et al. 2003). Parents were given the most extensive survey with 5 measures: (1) collective efficacy/social cohesion (Sampson et al. 1997), (2) socio‐emotional parenting scales (Moulton and Gehlbach 2021), (3) sense of safety (Mujahid et al. 2007), (4) crime (NEWS‐Y) (Rosenberg et al. 2009), and (5) adult behavior (SDQ18+) (Goodman et al. 1998). Throughout the program, community members also completed surveys to gauge their concerns about the causes of Bessemer issues and their solutions. Questions included demographics as well as Likert‐style questions about perceptions on crime, solutions, and programs.

Surveys were administered (pre‐intervention and post‐intervention) for the three groups (children, youth, and parents) to measure possible group changes in the Fall 2022 cohort and Spring 2023 cohorts for the outcomes listed above. All survey questions were presented in paper format, and researchers transferred the responses into an electronic database. Study data were collected and managed using REDCap (Research Electronic Data Capture) electronic data capture tools (Harris et al. 2019; Harris et al. 2009), hosted at the University of Alabama at Birmingham. The data was then verified for accuracy with two other researchers. Community coalition members were also given the opportunity to attend community meetings (citizen town halls) to discuss the progress. Community surveys were conducted at all community meetings to measure sense of safety (Mujahid et al. 2007) and collective efficacy (Sampson et al. 1997).

**Focus Groups.** Focus groups provided data from both program participants (parents and students) and community partners (implementers). Participants related their experiences and perceptions about the interventions. Focus group guides were designed to ask questions about the participant's experiences on the general program (reasons for participation, program and session details, behavior changes/program impact, and recommendations.

## 3 Results

### 3.1 Implementation

The intervention was implemented in 2022–2023 in partnership with 13 Bessemer area schools and community organizations. Community participants were recruited through grassroots community communications, as was done with the EAP. Intervention activities included summer camps and after‐school programming for children and youth and their guardians. Community members participated in activities such as community gardens, neighborhood association planning meetings, and citizen town halls. The primary site for intervention activities was a center located within one of the housing communities and the Bessemer Recreation Center.

Summer camp 2022 had the largest participation rate, with 500 students enrolling and an average of 267 students attending weekly. After‐school programs and summer camp 2023 had a lower participation rate, with approximately 40 students participating in each cohort. Changes in partnerships contributed to this participation rate change.

### 3.2 Process Data

Table 3 presents the **r**each of the BBB intervention (2022–2023) by SEM level (individual, relationship, community, and society). It also summarizes the components used in the multi‐component intervention. As the table shows, there was a wide range of residents, parents, children, and youth who participated in the activities.

**Table 3:** Summary of NOBLE implementation interventions, participants, and evaluation measures (2022–2023).

Intervention	Years	Participants (*N*)	Assessment measures (*N*)
Individual Level			
Summer Camp	2022	267^a^^,^ b	3 Focus groups (24 Elementary)
	2023	32^a^^,^ b	Survey (26 Elementary/Middle)
After School Program	2022	36^a^^,^ b ^,^ c	Survey (7 High)
	2023	55^a^^,^ b ^,^ c	2 Focus groups (11 Middle/High) Survey (35 Elementary/Middle/High)
Relationship Level			
Youth/Young Adult Mentoring	2023	42^a^^,^ b ^,^ c	—
Parent SEL Support	2022	17^d^	1 Focus group (8) Survey (12)
	2023	30^d^	2 focus groups (19) Survey (26)
Coordinator Focus Groups	2022	3	2 Focus groups (8)
	2023	5	
Community Level			
Police Outreach: TLYC	2023	30^a^^,^ b ^,^ c	Knowledge‐based survey provided by NOBLE (11 Elementary/Middle)
Community Service Projects	2023	24^a^	Two community sites
Community Gardening	2023	97^e^	3 community gardens established
Community Mural Assessment	2023	61^e^	Mural survey, site analysis
Arts Showcase	2023	65^e^	—
Society Level			
Neighborhood Assoc./Coalition Mtgs (2023)	March	16^e^	Satisfaction/Neighborhood survey Social Cohesion/Collective Efficacy (110)
	May	23^e^	
	July	71^d^^,^ e	
Citizen Town Halls	2022 2023	167^e^	

^a^ Elementary students.

^b^ Middle school students.

^c^ High school students.

^d^ Parents.

^e^ Community residents.

### 3.3 Outcome Data

Participants of various intervention components were not required to participate in the evaluation component.

### 3.4 Demographics

Adult and youth participants (*N* = 102) provided their demographic data at the time of the program survey. Demographic questions included sex, race, and ethnicity. Of the 38 adults completing the surveys, 95% (*n* = 36) identified as female, while 5% (*n* = 2) identified as male. Of the 38 adults, 92% (*n* = 35) identified their race as Black, with the remaining (*n* = 3) identifying as non‐Black. 11% (*n* = 4) identified as Hispanic ethnicity. Of the 64 children/youth student participants who completed the demographic questions (elementary, *n* = 26; middle school, *n* = 22; high school, *n* = 16), 100% identified as Black, with 47% female and 53% male.

Table 2 also presents the intervention evaluation outcomes for qualitative and quantitative assessments. This includes the data for surveys, focus groups and final evaluation outcomes.

### 3.5 Program Surveys

Program surveys (pretests and posttests) were only administered to participants who consented during the Fall 2022, Spring 2023, and Summer 2023 cohorts, as part of the project evaluation. Participants (youth and guardians) were recruited out of the pool of those attending programs implemented, for focus groups (Summer, Fall, and Spring), and for surveys (Fall and Spring). Students self‐selected into the program and evaluation and could opt out of the survey and focus groups if they so indicated. Not all program participants participated in the evaluation.

Program survey measures included:

*Social Cohesion/Collective Efficacy*. Findings indicate that social cohesion increased among youth (*N* = 38) (*p* = 0.009, Cohen's d = 0.47), as measured by the UNICEF pre‐post responses. No significant changes were seen in children or parent outcomes.

*Behavior (SDQ/Panorama)*. Improvements in self‐reported individual prosocial behaviors were seen in youth, with significant decreases in total difficult problems [(*p* < 0.003 and a moderate effect size (Cohen's d = 0.60)] and in conduct problems [(*p <* 0.001), a high effect size (Cohen's d = 0.86)]. No other significant changes in pro‐social were seen in youth, and children and parents showed no significant change in their SDQ scores (perceptions of child behavior).

*Crime/Safety NEWS‐Y (crime questions)*. A significant difference in perceived safety (*p* = 0.003; moderate effect size (Cohen's d = 0.63) was found among youth, indicating that youth felt more unsafe at post‐intervention. No changes were seen with children or parent outcomes for safety.

### 3.6 Community Surveys

Community surveys were administered at the end of events, such as coalition meetings, neighborhood associations, and citizen town halls. Community surveys included satisfaction questions as well as perceptions of neighborhood characteristics (Mujahid et al. 2007).

By the final community event (Fall 2023), community members (*N* = 110) completed community surveys, and for community safety, about 25% of respondents indicated they felt unsafe in their neighborhoods. 52% of community members agreed they felt safe walking in their neighborhood. 37% reported that their neighborhood was safe from crime, while 25% indicated violence was a problem in their neighborhood. Compared to previous community survey results (in the formative stages of the intervention), these values indicate community members may have perceived an increased level of safety following the intervention period.

### 3.7 Crime Rates

The evaluation plan included the collection and comparison of pre‐ and post‐intervention violent crime rates. However, while the Bessemer Police provided violent crime statistics at pre‐ and post‐intervention, accurately reporting and interpreting is difficult due to methodological inconsistencies in data collection, such as the interruption in reporting time frames due to the pandemic and nationwide changes in the methodology of reporting crime statistics to the FBI (FBI 2026). Due to such methodological issues, we hesitate to provide anything other than description of those results: when we observationally compare BPD‐reported violent crime data to national FBI rates, Bessemer violent crime post‐intervention appear to be on a downward trend, reflecting national crime trends for the same period (2026).

**Program Focus groups**. Focus groups (*N* = 7) were conducted at the end of each program implementation between Fall 2022 and Summer 2023—Program Coordinators/Community Partners (*N* = 2); Youth (*N* = 2); Parent/Guardians (*N* = 3). All focus groups were done in‐person at the Bessemer Recreation Center, the primary site for intervention activities. Focus groups were audio recorded, transcribed, and coded according to thematic analysis.

***Parent/Guardian Focus Groups***. Focus group findings indicate overall support of the programming among parents/guardians.

*Topics*. Praise was given by parents/guardians for SEL topics like understanding and naming emotions, mindfulness, resolving conflict at home, and preventing bullying. One mother described her learning as, *“…four types of bullying. I thought there was just one type. Four! I enjoyed that lesson. The only thing I regret, that it wasn't people – more people hear to hear these sessions.”* Another parent described how she took bullying knowledge back to her daughter: *“I had to take some of those ideas and sessions and topics and the knowledge that I learned here, take it home and talk to her about it and let her know, let somebody know if somebody's bullying you.”*

Parents/guardians expressed appreciation for real‐world examples applicable at home. One mother highlighted this applicability by recounting a home experience, *“I think my husband reminded me, because I go home every week and just talk about what we did, and so we were out of town, and something triggered me, and he said, ‘Don't forget your SEL strategy,’ so we were able to use it.”* Some of the programs included hands‐on activities to allow parents to practice their newly learned skills, like this parent explained, *“I liked the opportunity just to engage in role playing because I'm more tactical and just like to get involved hands‐on, and it just made my learning better.”*

*Program acceptability*. Parents/guardians spoke about the benefits of both social support and applicability. Social support was a strongly expressed sentiment, with one mother noting: *“this group has become part of a safe haven for me ‘cause there are certain things I might not want to express to people that I'm close to.”* The atmosphere was also one that set the parents at ease, as another mother recalls her experience:

*“When I came here, it was so much love. It was a no‐judgment zone. It was informative. It was everything you could imagine being under the stress of bein’ a single parent.”*

*Recommendations*. Parents/Guardians requested more sessions, with parents wanting to meet twice per week and lengthen the sessions. One mother suggested multiple partnerships to better promote the parent program, *“…the Bessemer Housing Authority, there are some good people there, and they try their best to provide things for people. Let people like that know about this [program]. Let people like the Board of Education in Bessemer know about it.”* Finally, parents/guardians requested greater accessibility, with some parents requesting a remote option for those who could not attend in‐person sessions.

***Youth Focus Groups***. Focus group findings also indicated overall support of the programming content by youth. Among the activities presented in the program, meditation as a stress reliever was a popular option, as was art therapy. One student described the benefits of learning meditation: *“I remember you taught us to close our eyes and meditate. You taught us to draw how we feel. That kind of helped relieve, I'm gonna say stress, stuff that you got goin’ on to get right. It helped that.”* Learning how to communicate was also beneficial, as one student recalls, *“I learned how to talk to people better instead of just blowin’ them off, just talk to them and explain myself.”*

Youth discussed their perceptions about the venue and activities, and generally felt that the community center setting was ideal as it was smaller, at a walkable distance and allowed the students to focus on the program. Students did note that the recreation center might be distracting because other children might be playing around them. Youth indicated that they would participate again, and there were several comments regarding having other after‐school options and choosing this program again

Youth felt that they would have preferred individual mentoring and not group mentoring, with some students choosing not to attend on days mentoring was scheduled. One middle school student commented, *“It was disappointing. Like they tried to get us to express our feelings and stuff. There were too many people in there.”* Another student expressed the role of peer pressure and embarrassment played, saying “…it was uncomfortable, especially with my cousin there.” Students also requested more outdoor activities. Several expressed the desire to have outdoor time after school since they stayed inside all day.

***Program Coordinators and Community Partner Feedback***. Overall, the perceptions and feedback of program coordinators and community partners who provided support for sessions during the SEL program indicated that the program was received well, with several partners continuing to work together to offer joint programs to kids and parents after the grant period was over. Some examples include a Bullying Summit in October 2023 (UAB partnered with Lions Quest and Brother, Let's Talk) and the continued partnership between Abundant Fountain of Life and The Cohill Foundation to continue offering art therapy for kids in a neutral space.

## 4 Discussion

This study reports the results of the design and implementation of an intervention, *Building a Better Bessemer: Innovations in Community‐Based Crime Reduction*, a holistic multi‐sector project, co‐designed with the community, to improve health, increase quality of life, increase collective efficacy, and reduce violent crime at the individual, relationship, community and society in a small city with a high crime rate. Using an iterative process with the community's input and feedback, we developed an intervention to address youth, parents, police, and community needs. This iterative process of community collaboration included multiple points of dissemination of findings and knowledge to all partners at early, mid and late stages of the implementation (Israel et al. 2017).

Community support was essential for the intervention design and implementation. Community partners co‐designed the Action Plan, helped recruit participants, and implemented programming. The Bessemer BHC Coalition assisted greatly with recruitment, and the coalition members were aware of neighborhood families who could benefit from the intervention. Coalition members also played a pivotal role in welcoming the research team, as members would distribute recruitment materials (i.e., flyers) at local events and would invite the research team to recruit at local events. Coalition members also assisted with program materials, such as donations of school, party, and garden supplies, etc. and volunteered when their schedule allowed. The final outcomes of this project were presented at a town hall, open to the community and their feedback.

Our intervention evaluation was implemented through surveys and focus groups, and overall, the results seem to indicate that the intervention is feasible and may benefit adolescents, as seen in increased collective efficacy outcomes and decreased negative behavior outcomes. Taken as a whole, these results provide us with optimism that a community‐engaged approach to addressing community violence is feasible and may address contributors and solutions to crime.

Our mixed methods evaluation found that BBB individual‐level programs led to significant decreases in negative behaviors and increased social cohesion in middle‐ and high‐school students. One of the most important outcomes was the generated momentum to improve community safety in the city, as seen in the community feedback survey results. While these non‐validated surveys were provided to gain the perceptions of the community, they may indicate that some momentum was generated by the intervention to improve community safety, even while more work remains to be done to increase community safety. The project also received widespread local support from the Mayor's office, the City Council, the Bessemer BHC Coalition, the Bessemer School Board, and other community organizations. This community investment indicates the increased value that the Bessemer community placed in the collaborative BBB intervention.

Findings indicate that our intervention may have increased positive outcomes in perceived social cohesion among youth, in line with other community safety and community cohesion studies (Reese et al. 2001). One multi‐level intervention for urban youth found increases in social cohesion scores from pre‐ to post‐intervention, similar to this study (Berg et al. 2009). Results for altering perceived adult social cohesion levels have been less successful in community‐based interventions (Ohmer 2016), as was the case in the current intervention findings. Our lack of adult social cohesion outcomes may also be a result of the limited sample. Parents/guardians were recruited for surveys through the program, which may have affected these results. Additionally, they may not have received enough exposure to the intervention components that would have resulted in changes in social cohesion outcomes.

Our SEL program post‐test results indicate that youth perceived significant decreases in negative behavior and increases in prosocial behavior following the intervention. Previous intervention studies document the benefits of incorporating life skills education to promote the reduction of delinquent conduct and the development of social skills (Gargan 2017; Holahan and Batey 2019; Matischek‐Jauk et al. 2018). Youth interventions incorporating behavioral components report increased prosocial and decreased aggressive behavior scores among urban youth (Zimmerman et al. 2018), with emotional and social intelligence abilities being a predictor of psychological adjustment for youth exposed to community violence (Hawkins 2012; Stokes and Jackson 2014). Additionally, our SEL education component of the intervention targeted both caregivers and youths, both factors in community violence studies (Fagan and Catalano 2013; Ross et al. 2021).

Interestingly, our results indicated that the youth perceived themselves to be more unsafe following the intervention. Other intervention studies have found similarly mixed results (Dodington et al. 2012; Project 2008), with violence interventions increasing risk awareness not previously considered. Our focus group results indicated that participants generally approved of the intervention, in line with previous intervention studies asking feedback of youth, caregivers, and stakeholders (Dodington et al. 2012). Additionally, the Bessemer violent crime rate trend during the intervention appears to reflect the national downward crime trend. Taken as a whole, these outcomes provide us with careful optimism that a community‐engaged approach to addressing community violence can address contributors and solutions to crime.

Limitations to this pilot study include its limited generalizability due to the number of participants completing the evaluation and the fact that the designed intervention was location‐specific, for the needs of a specific community. Though this is a feasibility project, one limitation of the study is the ratio of respondents to participants, and with few survey responses to many participants, we acknowledge there is the potential for selection bias. Additionally, it can be difficult getting participants to complete evaluation forms. Future studies should anticipate how to address such potential attrition when addressing programming. Care should be taken when interpreting these results, and they are not generalizable.

Another limitation of this study is that there are no measures of “dosage” or “exposure” available for the participants. It is possible that students who participated in the 2022 summer school also participated in the Fall and Spring afterschool 2023 cohorts. While verification of registration data confirms that no participants had duplicate registrations across the afterschool participants, it is unclear how much program exposure participants had with the summer camp. Without this information, it is difficult to account for the level of exposure to the intervention and how that affects the outcomes. However, the community‐engaged IM **process** we utilized to develop and evaluate the BBB intervention could be generalizable to other communities.

Further, although our participants desired that we implement societal‐level changes, such as addressing poverty and deficiencies in the public schools, we did not have the resources to address such factors. While addressing systemic influences was beyond the scope of this study, systemic influences can strongly shape local capacity for violence prevention through funding trends, policy design, and governance structures. This is especially relevant in the funding status for community violence prevention projects. For example, restrictive eligibility criteria and evidence standards embedded in federal statutes such as the Family First Prevention Services Act [FFPSA] unintentionally limit access to critical services in under‐resourced jurisdictions (Stoltzfus 2018). Similarly, abrupt federal grant terminations—such as the 2025 withdrawal of over $800 million in Office of Justice Programs awards—illustrate how volatility in national priorities may undermine continuity of community‐based interventions (Kemper and Finklea 2025). These disruptions may not only constrain program implementation but may also erode trust among local stakeholders, weakening collaborative momentum.

From a social‐ecological perspective, these systemic shocks could reverberate across all levels of the model, influencing organizational stability, community cohesion, and ultimately individual outcomes. These conditions underscore the need for interventions that address not only individual and community factors but also structural determinants (Sampson and Wilson 1995; Sampson et al. 1997; CDC 2024). Addressing youth violence, therefore, requires strategies that extend beyond programmatic design to include advocacy for durable policy commitments, equitable resource allocation, and mechanisms that insulate prevention efforts from political and fiscal instability. Embedding these considerations into intervention frameworks strengthens sustainability and aligns calls for structural competency in public health (Bronfenbrenner 1979; Farrington and Ttofi 2020).

## 5 Conclusion

Despite limitations, by engaging the community in an iterative IM process, we were able to develop a holistic, multi‐level intervention to address community violence that is community‐owned and supported. *Building a Better Bessemer* equitably involved partners in determining problems and solutions related to violence as an SDH and ultimately offered alternatives to participating in crime for youth, programs to support positive child and parent opportunities, events to encourage interactions between the community and law enforcement, and activities to foster structural and systemic change at the community level to increase the safety, well‐being, and health. And, because local organizations were engaged in implementing the intervention, programs are ongoing in the community, potentially leading to sustainable change.

## Author Contributions

**Catanya G. Stager:** conception and design, analysis and interpretation of data, drafting the manuscript, revising manuscript critically for important intellectual content. **Samantha Whitfield:** acquisition of data, drafting the manuscript, analysis and interpretation of data. **Ariann Nassel:** acquisition of data, analysis and interpretation of data, revising manuscript critically for important intellectual content. **Henry Irby:** conception and design, revising manuscript critically for important intellectual content. **Mike Wood:** acquisition of data. **Tiffany Osborne:** conception and design, acquisition of data, revising manuscript critically for important intellectual content. **Grace Okoro:** acquisition of data, revising manuscript critically for important intellectual content. **Melissa Thompson:** conception and design, revising manuscript critically for important intellectual content. **Penny Willis:** conception and design, revising manuscript critically for important intellectual content. **Dwayne A. Crawford:** conception and design, acquisition of data revising manuscript critically for important intellectual content. **Matthew Kiefer:** conception and design, revising manuscript critically for important intellectual content. **Erin Carley:** acquisition of data, analysis and interpretation of data. **Christson Adedoyin:** analysis and interpretation of data, revising manuscript critically for important intellectual content. **Yu‐Mei Schoenberger:** analysis and interpretation of data, revising manuscript critically for important intellectual content. **Lori B. Bateman:** conception and design, acquisition of data, analysis and interpretation of data, drafting the manuscript, revising manuscript critically for important intellectual content.

All authors have provided final approval of the manuscript to be published. Each author has participated sufficiently in the work to take public responsibility for appropriate portions of the content. All authors have agreed to be accountable for all aspects of the work in ensuring that questions related to the accuracy or integrity of any part of the work are appropriately investigated and resolved.

## Ethics Statement

This research was approved by an institutional research ethics board of the University of Alabama.

## Consent

Informed consent was obtained from all participants in this study.

## Conflicts of Interest

The authors declare no conflicts of interest.

## Data Availability

The data that support the findings of this study are available from the corresponding author upon reasonable request. The data is not publicly available due to privacy and ethical restrictions.
